# Diseases of Women and Children

**Published:** 1859-07

**Authors:** Emil Fischer


### DISEASES OF WOMEN AND CHILDREN.

BY EMIL FISCHER, M.D.

### I.—*New Theory and Treatment of Chlorosis.* By DR. VON MAACK.

The glycogenic function of the liver is hardly made known, and already pathologists hasten to assign to it a part in the pathogeny of diseases. Although we receive the ideas of the author with the greatest reserve, we republish his theory in order to illustrate this scientific tendency, common to all ages.

"The diminution of the red corpuscles in the blood of chlorotic patients," says the author, "is an established fact. These corpuscles owe their color to the iron which they contain; it is, therefore, evident that the quantity of this metal in the blood is diminished in cases of this kind. This diminution is not the result of resorption, for the urine contains only a small quantity of solid matter, but it is the consequence of bad elaboration.

"We know also that the very small quantity of iron which the organism in the healthy state draws from the ingested aliments is quite sufficient for all its wants, and that the bile is the only secretion which contains it in notable quantity.

"This being known, how do we explain the formation or development of chlorosis in a young woman who enjoyed good health previously? She takes the same aliments after the appearance of the disease as before; it is, therefore, impossible to attribute this affection to a privation of the ferruginous element, for, afterwards, as before, the same quantity of it is absorbed, and has sufficed for many years to maintain health.

"The real cause is more likely this, that it is impossible for the organism to transform the iron into hematin and to fix it. From what does this impossibility arise? M. Lehmann has proved that hematin, like salicin, phloorrhizein, etc. is a saccharine compound. Hematin needs, therefore, sugar for its formation. Thus, as soon as the saccharine secretion of the liver is diminished or arrested, the formation of the coloring substance of the blood will cease, and consequently that of the red corpuscles.

"The true origin of chlorosis would, consequently, be the want or the diminution of the quantity of sugar elaborated by the liver."

The author draws the following conclusions from his premises:—

1. The treatment of chlorosis must consist in the use of sugar. 2. The object of the treatment must be to re-establish the saccharine secretion of the liver. 3. The medication which consists in the abundant use of iron has not introduced into the organism an element which was wanted there, as is believed, but has cured by acting upon the healthy secretion of the liver.

The best remedy, according to Dr. Von Maack, consists in the use of grape-sugar and of honey. This treatment of chlorosis, it seems, has been practiced for a long time by the people in the northern part of Schleswig, and of certain regions of Hanover. An adjuvant to it is cold water used freely as a drink; already Petter has recommended it as excellent in diabetes. (*Archiv für Wissentschaftliche Heilkunde*, and *Union Médicale*, February, 1859.)

II.—*Memoir on the Hypertrophic Elongation of the Cervix Uteri.* By M. HUGUIER. (Presented to the *Académie de Médecine* of Paris, March 8, 1859.)

In a meeting of the *Académie de Médecine*, held on the 15th of March, M. Huguier read the principal passages of the last part of his memoir on the hypertrophic elongation of the neck of the womb. The following conclusions contain the substance of his treatise:—

1. Falling of the womb, be it complete or incomplete, is not a disease by itself, but rather an ensemble of several affections designated by a single name.

2. When the uterus protrudes externally, even then, when the vagina is completely inverted, and the womb, from the volume of the tumor, the centre of which it occupies, seems to be entirely prolapsed between the thighs, it is not, in the very great majority of cases, because the whole organ is fallen and has completely left the pelvis, but because it has undergone a partial or general hypertrophic elongation.

3. In the affection designated by the name of prolapsus, the hypertrophic elongation is not the exception, but the very general rule.

4. Two principal varieties of longitudinal hypertrophy, the *sub* and the *supra-vaginal*, which constitute, in some measure, two different diseases, may simulate procidentia and prolapsus uteri.

*First Part.* 5. In the first variety of elongation, the neck of the womb forms in the cavity of the vagina a cylindroid or conoid more or less elongated projection, the free extremity of which approaches the opening of the vulva, or protrudes even between the labia of the part, without the vulvo-uterine canal being shortened, invaginated, or inverted upon itself.

6. It has been until lately confounded with procidentia and prolapsus of the womb, if it was not mistaken and treated for a polypus, chronic inversion, a follicular cyst, scirrhus of the neck, or dropsy of this part.

7. A complete description of it has not yet been given, although it has striking characters with regard to its development, its symptoms, and its treatment.

8. Medicines, and the different varieties of cauterization, are only applicable to slight hypertrophy, and that complicated with inflammation and congestion.

9. Pessaries are mostly useless and dangerous.

10. When the hypertrophic elongation of the cervix uteri gives rise to serious symptoms, and if it has attained a length of five to seven centimeters, there is only one truly efficacious and curative measure to be employed—that is, resection of the neck, half a centimeter below the insertion of the vagina.

The author has reported in his treatise seven cases which corroborate this statement.

*Second Part.* 11. The disease designated, until the present, by the name of complete prolapsus, or falling of the womb, is generally nothing else but a longitudinal hypertrophy of the supra-vaginal portion of the organ, the body and fundus of which have remained in the cavity of the pelvis, although the vagina may be completely inverted, and the tumor hanging between the thighs may be of equal or greater length than the uterus in the normal state.

The accuracy of this proposition is proved by historical researches, pathological anatomy, and clinical facts.

12. The cases of hypertrophic elongation of the supra-vaginal portion of the neck, which we find here and there reported by the authors of the last two and the present centuries, have been left unnoticed, and were until the present time completely lost to science. The authors themselves have not drawn any practical conclusions from these cases, and have always confounded this affection with real prolapsus of the uterus.

13. There is scarcely any work in which we find the unexceptionable proof, by symptoms and pathological anatomy, that the complete prolapsus of the uterus exists.

14. On the contrary, the anatomico-pathological facts which we have described, those which several of our colleagues have, since our observations, demonstrated to the *Société de Chirurgie*, and those contained in the museum of Dupuytren, prove the frequency of hypertrophic elongation, and of the falling of the neck alone in the affection called prolapsus of the womb.

15. The longitudinal hypertrophy of the supra-vaginal portion of the neck, and the falling of the womb, have different pathological and semeiotic characters which serve to distinguish these two affections.

16. The relaxation, weakness, and forced distention of the large or the round ligaments, do not contribute in a really efficacious manner to the falling of the uterus any more than the destruction of these ligaments; such is not the case with the analogous alterations of the utero-lumbar (utero-sacral) ligaments.

17. In treating this affection, we should resort to a bloody, or properly so-called surgical operation, only when it gives rise to serious symptoms, and we are certain that medical and prothetic means are insufficient.

18. All the operations which have been invented up to this day, in order to fulfill the therapeutic indications of the disease, are insufficient. They can be useful in the case of simple falling of the uterus without hypertrophic elongation, and in this respect should not be discarded from science.

19. In the hypertrophic elongation of the neck, which is followed by prolapsus of this part and by inversion of the vagina, the only operation which fulfills the principal indications, and may be followed by success, is the amputation of the neck above the insertion of the vagina, more or less near the body of the organ, according to the degree of the elongation.

20. This operation must never be performed without precautions against the consecutive inflammation having been previously taken. These precautions must be carefully continued for fifteen or twenty days after the operation.

21. The arteries of the uterine tissue are seized and ligated with great difficulty; in order to do it promptly and surely, it is necessary to use a kind of tenaculum, which is to be left in place until it falls spontaneously.

22. The *écraseur linéaire* has appeared to us useful for terminating the section of the neck, particularly if this part is very vascular.

23. If the disease is preceded by a rectocele or a voluminous cystocele, or by both affections at the same time, it may be necessary, after having amputated the neck, to operate separately on the hernia of the rectum, and of the bladder, as we have done several times with success.

24. The operation is contra-indicated, if there exist at the same time a very large pelvis and vulvar opening, a more or less torn perineum, and a considerable weakness of all the soft parts which form the floor of the pelvis.

25. If we do not operate under conditions indicated in the preceding conclusion, the disease is not liable to a relapse, and the health of the patient becomes as good as it had been before the development of the affection. (*Gazette Hebdomadaire*, March 18, 1859.)

### III.—*On the Origin of Flexions of the Uterus.* By PROF. VIRCHOW.

Virchow sums up his very elaborate treatise on the mode in which flexions of the uterus originate, in the following propositions:—

In the history of flexions we may distinguish three different periods: one of mere predisposition, one of simple flexion, and one of flexion complicated with different inflammatory processes.

The predisposition is frequently created by partial peritonitis; it manifests itself by attacks of the nature of colic, and is at least somewhat lessened by early attention.

Long-continued retention of the urine and of the fæces favors the formation of flexion, particularly at the time of menstruation, the puerperal state, etc., and is, therefore, to be carefully avoided. Enlargements of the uterus, particularly if connected with relaxation of the organ, can increase the flexion very rapidly, while the removal of these conditions, for instance in chronic endometritis, may diminish the flexion to a considerable degree. It is, therefore, of great consequence to keep careful watch over the menstrual and puerperal periods, and to allay catarrhal inflammation of the uterus by antiphlogistic treatment and other means.

It is very questionable whether antiflexion can be completely removed; in retroflexion a cure may be attempted. If the flexion is complicated with consecutive affections, particularly with endometritis or perimetritis, a persistent and careful local treatment is requisite. Endometritis may be removed by it; perimetritis, on the contrary, produces adhesions of the uterus, which confirm the flexion more and more the longer they exist, and render it almost impossible to correct the position of the organ.—(*Allgemeine Wiener Medizinische Zeitung*, 1859, 6.)

### IV.—*On Inflammation of the Fallopian Tubes as a Cause of Puerperal Peritonitis.* By PROF. E. MARTIN.

In one of the meetings of the *Gesellschaft für Geburtskunde* of Berlin, Prof. E. Martin gave a very interesting lecture on inflammation of the Fallopian tubes as a cause of puerperal peritonitis. An experience of many years has convinced Prof. Martin that—with the exception of severe injuries of the genitals during the act of labor, which may cause the death of the patient in different ways within the first few days—the most common cause of death among lying-in women is pyæmia and emboly, which may be either produced by thrombosis of the veins of the uterus and of the vagina, or by the reception and generation of pus in the lymphatic vessels of the genitals, and which give rise to fever and a great variety of symptoms.

Besides this kind of puerperal disease, the author has, however, observed another less frequent, but just as fatal a one, which depends upon *a propagation of the endometritis into the Fallopian tubes, and an effusion of the purulent products of this salpingitis into the peritoneal cavity.* This effusion of the contents of the Fallopian tubes into the cavity of the abdomen is followed in most cases by fatal peritonitis. In spite of the open abdominal end of the tubes, their contents are not generally discharged at once into the peritoneal cavity, but only after some unusual movement, etc., during which the abdominal muscles contract and press upon the tubes; this is easily explained by the fact that the external portion of the canal of the tube enlarges itself into a sinus as soon as fluids accumulate within the tube. According to the author's opinion, the metrosalpingitis observed in lying-in women does not always originate first during confinement, but sometimes during pregnancy, and occasionally even previous to that. One of the principal circumstances which speak for the long existence of the catarrhal inflammation of the tubes, is the considerable enlargement and textural change of the canal, as the author has observed it in all his cases in which a post-mortem examination was made; also in unimpregnated women Prof. Martin has observed this affection; it was then the consequence of a metritis produced by a gonorrhœal infection; after the painfulness of the vaginal portion had disappeared, and nothing remained but a muco-purulent discharge, a lively pain was again felt in the depth of one or the other inguinal regions (without there being any swelling of the adjacent ovary perceptible;) this pain was increased on pressure; it generally yielded to the application of leeches cataplasms, and tincture of iodine. As adhesions of the tubes to neighboring organs are a post-mortem appearance, frequently found in prostitutes, there is good reason to assume that salpingitis often occurs in consequence of sexual excitement, particularly if the latter is combined with infection.

As regards the symptoms of metrosalpingitis, the principal one, in the acute form of the disease, is pain; this is of pretty intense character, and is felt in both inguinal regions, if the disease exists on both sides, as it usually does. The seat of the pain offers the principal reasons for suspecting the presence of the disease, and, by resorting to the method of exclusion, this diagnosis may be rendered more probable. Sometimes it is possible to feel on external, but particularly on internal, exploration, the swelled and enlarged tubes in the form of oblong rolls. The disease under consideration requires, however, during the first days of confinement, the greatest precaution in making palpation, as each strong pressure is connected with the danger of effusion taking place into the peritoneal cavity; it is, therefore, often necessary to be satisfied, under these circumstances, with an only conjectural diagnosis.

The prognosis of metrosalpingitis in lying-in women is not unfavorable as long as the products of inflammation remain in the cavity of the tube, as a part of them may be removed by absorption, while the rest becomes concrete; there are also, in the generality of cases, adhesions formed between the respective tube and the neighboring organs. Both these changes are generally followed by sterility. In connection with this subject it is well not to overlook the possibility of the products of exudation undergoing tubercular degeneration; in fact, tubercular masses are more frequently met with in the Fallopian tubes than in any other part of the female organs of generation. Sometimes, when the abdominal opening of the tube has been closed by adhesions, dropsy of the tube follows. With all this, life may continue for a long time, although it may be accompanied by various troubles, arising principally from the incurable changes of form and position which the internal genitals have been subjected to. Sometimes, finally, after the dilated tube has formed adhesions with the abdominal parietes, its secretion may be discharged externally, and thus health may be restored to some degree. If the contents of the dilated tube are effused into the peritoneal cavity the preservation of life seems to be possible only when the secretion is not of a purulent, but of a mucous character, and when the consequent peritonitis remains confined to a certain locality. Of this the author has convinced himself in several cases, in which, on the post-mortem examination of individuals who had suffered, many years before their death, from puerperal peritonitis, membranous adhesions of the tube and of the ovary were found confined to only one-half of the cavity of the pelvis.

The treatment of metrosalpingitis in lying-in patients must be antiphlogistic; the principal condition for recovery is, of course, long-continued rest in one position, and the avoidance of every movement requiring exertion.—(*Monatsschrift für Geburtskunde*, 1859, XIII. 11.)

V.—*Observations on the Treatment of Diphtheritic Angina and Laryngitis in Children.* By DR. HAUNER.

Both diseases occur only in feeble children, debilitated by previous diseases, mostly, however, in such who have suffered either recently or some time previously from an exanthematous disease. Diphtheritis is contagious, and can be transmitted from sick to previously healthy children; it may become fatal by general infection, pyæmia, or loss of strength, as well as by propagating itself to the larynx, bronchi, and lungs. That treatment will alone be successful which endeavors to stop the progress of the diphtheritic process to important organs, anticipating it, as it were, by local means, and which tries to strengthen the constitution by internal remedies. The antiphlogistic treatment is decidedly obnoxious. Of all local remedies the author considers nitrate of silver the most efficient; he applies the caustic in substance to the diphtheritic layers in the mouth, on the uvula, throat, etc., and takes care to carry the application somewhat beyond the diseased surface. Instead of the stick, a strong solution of nitrate of silver (℈j–ʒss to ℥j of distilled water) may be used. In regard to the prophylactic value of this local treatment the author observes, that he has not seen a single case of diphtheritis of the mouth and throat in which the disease spread and reached the larynx after having been thus treated. But even in diphtheritic croup nitrate of silver is useful in combination with the internal treatment; the application is, in this case, made by means of a whalebone probang having a pencil of charpie attached to its extremity, or finely-powdered lunar caustic (gr. iij–iv) quickly blown in through a quill, the tongue being depressed by a spatula. In some cases the author precribed, at the beginning of the disease, an emetic of ipecacuanha, and found it decidedly useful. In regard to the internal treatment of diphtheritis, it is particularly necessary to enjoin a generous diet, (good broth, Liebig's extract of meat, coffee, beer, wine, etc.) The best internal remedy is quinia, given with alternate doses of chlorate of potassa, (ʒss–j to ℥iij–iv of distilled water;) the latter remedy exerts a very favorable influence upon the local diseases of the mouth and throat. A useful adjuvant in the treatment of diphtheritic angina and laryngitis is the local application of moist heat by means of compresses soaked in water, and covered over with a dry linen cloth, or other material to retain the heat and moisture. The author finally cautions against the treatment of diphtheritic croup by bleeding, mercurial ointment, calomel, tartar emetic, etc. By making a careful examination of the patient, and by taking the constitution and the previous history of the child into consideration, it will not be difficult to distinguish diphtheritic from true laryngeal croup. — (*Œsterreichische Jahrbücher für Kinderheilkunde*, 1859, II. 2.)

